# Health literacy among people with systemic sclerosis: a Scleroderma Patient-Centred Intervention Network (SPIN) Cohort cross-sectional study

**DOI:** 10.1093/rheumatology/keag558

**Published:** 2026-10-19

**Authors:** Claire Adams, Marie-Eve Carrier, Meira Golberg, Natalie Co, Elsa-Lynn Nassar, Linda Kwakkenbos, Susan J Bartlett, Catherine Fortuné, Amy Gietzen, Geneviève Guillot, Amanda Lawrie-Jones, Vanessa L Malcarne, Maureen D Mayes, Michelle Richard, Luc Mouthon, Andrea Benedetti, Brett D Thombs, SPIN Investigators, Benjamin Chaigne, Karen Gottesman, Sabrina Hoa, Marie Hudson, Laura K Hummers, Yanne Perriault, Danielle Rice, Maureen Sauvé, James Stempel, Robyn K Wojeck, Christian Agard, Laurent Alric, Marc André, Floryan Beaslay, Elana J Bernstein, Sabine Berthier, Lyne Bissonnette, Sophie Blaise, Alessandra Bruns, Carlotta Cacciatore, Patricia Carreira, Marion Casadevall, Lorinda Chung, Benjamin Crichi, Thylbert Deltombe, Christopher P Denton, Tannvir Desroche, Robyn Domsic, James V Dunne, Bertrand Dunogue, Regina Fare, Dominique Farge, Paul R Fortin, Tracy Frech, Loraine Gauzère, Anne Gerber, Jessica K Gordon, Brigitte Granel, Aurélien Guffroy, Geneviève Gyger, Eric Hachulla, Daphna Harel, Monique Hinchcliff, Michael Hugues, Alena Ikic, Sindhu R Johnson, Nader Khalidi, Kimberly S Lakin, Marc Lambert, Maggie Larche, David Launay, Yvonne C Lee, Paul Legendre, Catarina Leite, Hélène Maillard, Nancy Maltez, Joanne Manning, Marie Isabelle, Maria Martin Lopez, Thierry Martin, Ariel Masetto, Arsène Mekinian, Sheila Melchor Díaz, Morgane Mourguet, Christelle Nguyen, Karen Nielsen, Mandana Nikpour, Louis Olagne, Vincent Poindron, Janet Pope, Susanna Proudman, Grégory Pugnet, Loïc Raffray, François Rannou, Alexis Régent, Frederic Renou, Sébastien Rivière, David Robinson, Esther Rodríguez, Tatiana Sofia, Sophie Roux, Perrine Smets, Vincent Sobanski, Robert F Spiera, Virginia Steen, Evelyn Sutton, Carter Thorne, Damien Vagner, John Varga, Pearce Wilcox, Vanessa Cook, Cassidy Dal, Djellza Dani, D Monica, Sophie Hu, Nicole Iordache, Maria Nassar

**Affiliations:** Lady Davis Institute for Medical Research, Jewish General Hospital, Montréal, Québec, Canada; Department of Psychiatry, McGill University, Montréal, Québec, Canada; Lady Davis Institute for Medical Research, Jewish General Hospital, Montréal, Québec, Canada; Lady Davis Institute for Medical Research, Jewish General Hospital, Montréal, Québec, Canada; Lady Davis Institute for Medical Research, Jewish General Hospital, Montréal, Québec, Canada; Department of Psychology, McGill University, Montréal, Québec, Canada; Lady Davis Institute for Medical Research, Jewish General Hospital, Montréal, Québec, Canada; Department of Psychology, McGill University, Montréal, Québec, Canada; Department of Clinical Psychology, Behavioural Science Institute, Radboud University, Nijmegen, The Netherlands; Centre for Mindfulness, Department of Psychiatry, Radboud University Medical Center, Nijmegen, The Netherlands; Department of Medicine, McGill University, Montréal, Québec, Canada; Research Institute of the McGill University Health Centre, Montréal, Québec, Canada; Ottawa Scleroderma Support Group, Ottawa, Ontario, Canada; Steffens Scleroderma Foundation, Albany, NY, USA; Sclérodermie Québec, Longueuil, Québec, Canada; Scleroderma Australia, Melbourne, Victoria, Australia; Scleroderma Victoria, Melbourne, Victoria, Australia; Department of Psychology, San Diego State University, San Diego, CA, USA; San Diego State University and University of California, San Diego Joint Doctoral Program in Clinical Psychology, San Diego, CA, USA; University of Texas McGovern School of Medicine, Houston, TX, USA; Scleroderma Atlantic, Halifax, Nova Scotia, Canada; Service de Médecine Interne, Centre de Référence Maladies Autoimmunes et Autoinflammatoires Systémiques Rares d’Ile de France, de l’Est et de l’Ouest, Hôpital Cochin, Paris, France; Assistance Publique Hôpitaux de Paris-Centre, Hôpital Cochin, Université Paris Cité, Paris, France; Research Institute of the McGill University Health Centre, Montréal, Québec, Canada; Department of Epidemiology, Biostatistics, and Occupational Health, McGill University, Montréal, Québec, Canada; Respiratory Epidemiology and Clinical Research Unit, McGill University Health Centre, Montréal, Québec, Canada; Lady Davis Institute for Medical Research, Jewish General Hospital, Montréal, Québec, Canada; Department of Psychiatry, McGill University, Montréal, Québec, Canada; Department of Medicine, McGill University, Montréal, Québec, Canada; Department of Epidemiology, Biostatistics, and Occupational Health, McGill University, Montréal, Québec, Canada

**Keywords:** cross-sectional study, patient-reported outcomes, systemic sclerosis, scleroderma, rare diseases, wider determinants of health

## Abstract

**Objective:**

Health literacy reflects the ability to find, understand, evaluate, and use information to make health-related decisions. We evaluated sociodemographic and disease factors associated with health literacy among people with SSc.

**Methods:**

Participants with SSc in the Scleroderma Patient-centred Intervention Network Cohort completed the Health Literacy Scale_19_ − 12 Item Questionnaire from February to November 2025. Multivariable linear regression was used to assess associations of sociodemographic and disease characteristics with health literacy.

**Results:**

The 1019 participants were mainly female (*N* = 898, 88%) and White (*N* = 850, 84%). Mean (SD) years of education was 15.5 (3.9). Most had limited cutaneous SSc (*N* = 669, 66%). Mean health literacy total score was 69.7; 438 (43%) participants had excellent or sufficient and 581 (57%) had problematic or inadequate health literacy. Education (1.63 points per 5 years, 95% CI 0.25–3.01) was significantly associated with higher health literacy scores. Compared with the United States, participants from Canada (−4.98 points, 95% CI −7.99 to −1.97), the United Kingdom (−7.79 points, 95% CI −12.03 to −3.55), France (−6.05 points, 95% CI −8.84 to −3.27), and Australia, Mexico or Spain (−7.02 points, 95% CI −12.39 to −1.66) had significantly lower health literacy scores. Age, gender, race or ethnicity, marital status, time since first non-Raynaud symptom onset and disease subtype were not associated with health literacy.

**Conclusion:**

The association between health literacy and education was small. Differences between countries may reflect self-evaluation tendencies. More work is needed to better understand health literacy measurement.

*Rheumatology* key messages

- Higher education had a statistically significant but minimal magnitude association with higher self-reported health literacy among people with SSc.
- Residing in the USA was significantly associated with higher self-reported health literacy, but this may be due to differences in self-reporting tendencies.
- Future research is needed to better understand how we define and measure health literacy.

## Introduction

Health literacy is defined as an individual’s ability to find, understand, evaluate and use information to make decisions about their health [1, 2]. Key components include (i) having knowledge of health, healthcare and healthcare systems; (ii) being able to process and use health and healthcare information; and (iii) engaging in self-management and effective interactions with healthcare providers [3]. Health literacy facilitates shared decision making, where people participate in two-way collaborative relationships with their healthcare providers and discuss care options in light of their values and preferences and physician knowledge and recommendations [4].

Health literacy and shared decision making are associated with positive health outcomes, including improved quality-of-care indicators, better patient-reported health and reductions in emergency department visits [5, 6]. In rare diseases, healthcare professionals may have limited experience with a disease and patient-oriented information may not be available, making health literacy and shared decision making critical [7]. Barriers to health literacy may include difficulty understanding health information due to its complexity and the use of medical jargon in available information sources [8]. This can result in low adherence to treatment, impaired shared decision making and poorer quality of life [9].

The relationship between sociodemographic characteristics and health literacy has been explored in general population samples and among people with medical conditions. Among the largest studies, a nationwide study in the USA (*N* = 14 592) [10] and a multinational study of eight European countries (total *N* = 8000) [11] reported that younger age, higher socioeconomic status and higher education level were associated with greater health literacy. Systematic reviews and large-scale epidemiological studies have found greater health literacy among people with chronic diseases to be associated with female sex, higher socioeconomic status and higher educational level [12–16]. Race or ethnicity may also be associated with health literacy [12, 16], although categorization of race or ethnicity and its meaning in terms of socioeconomic positioning is often context- or country-specific.

There is limited health literacy research in rare diseases, including among people with SSc. SSc is a rare autoimmune disease characterized by fibrosis of the skin and internal organs and vasculopathy [17]. We identified only two studies on health literacy in SSc. One study, conducted in China, reported on the development of an SSc-specific health literacy measurement tool [18], but the constructs used in the tool deviated from commonly used theoretical frameworks [1, 3] and included treatment compliance, for instance, which is not part of health literacy [18, 19]. The other study, which was conducted in Italy, included 57 participants and provided only descriptive analyses of participants’ sociodemographic and clinical characteristics and health literacy levels [20]. Factors associated with health literacy were not assessed [20].

In previous work [21], we investigated factors associated with eHealth literacy among 333 people with SSc in the Scleroderma Patient-centred Intervention Network (SPIN) Cohort. EHealth literacy differs from health literacy in that it measures people’s ability to find and use health information on the internet but not their general ability to understand, evaluate and apply information [22]. We found that eHealth literacy was not associated with age or education level, but was associated with country [21]. SPIN Cohort participants from the USA reported greater eHealth literacy than those from Canada; France; the UK; and Australia, Mexico, or Spain (statistically significant for all except the UK).

To our knowledge, no study has evaluated factors associated with health literacy in SSc. Thus, the objective of the present study was to identify sociodemographic and disease factors associated with health literacy among people with SSc.

## Methods

This was a cross-sectional study that evaluated data from participants in the SPIN Cohort [23–25]. Because methods of studies that use SPIN Cohort data are similar, we adopted some of the same methods as in previous studies and followed guidance from the Text Recycling Research Project [26]. We reported results following guidance in the Strengthening the Reporting of Observational Studies in Epidemiology Statement [27]. The protocol was published on the Open Science Framework prior to initiating the study (https://osf.io/6n8uj/files/xy5b9).

### Participants and procedures

The SPIN Cohort is a convenience sample of participants from seven countries: Australia, Canada, France, Mexico, the UK, the USA and Spain [23–25]. Eligible participants are recruited by an attending physician or nurse coordinator during regular physician visits at 51 SPIN sites. Participants included in the SPIN Cohort must be aged ≥18 years; fluent in English, French or Spanish; and classified as having SSc based on 2013 American College of Rheumatology/European League Against Rheumatism criteria [28] as verified by a SPIN site physician. After obtaining written informed consent from eligible participants, onsite staff submit an online medical data form and participants receive an automated email with instructions on how to activate their online SPIN account. Participants complete outcome measures via an online portal upon enrolment and subsequently every three months. The SPIN Cohort was approved by the Research Ethics Committee of the Centre intégré universitaire de santé et de services sociaux du Centre-Ouest-de-l’Île-de-Montréal (#MP-05–2013-150) and by the ethics committees of all recruiting sites. Participant recruitment is ongoing. The present study included data from participants who completed the Health Literacy Scale_19_ − 12 Item Questionnaire (HLS_19_-Q12) [29], which was administered in the SPIN Cohort from February to November 2025.

### Measures

SPIN Cohort participants provided sociodemographic data (race or ethnicity, gender, years of education, relationship status, country, housing location) and completed patient-reported outcomes at baseline and subsequent assessments. At enrolment, physicians reported participants’ age, sex and medical data, including years since initial onset of non-Raynaud phenomenon symptoms and SSc subtype (limited, diffuse, sine).

#### Health literacy

The HLS_19_-Q12 [29] is a short version of the HLS Europe 47 Item Questionnaire (HLS-EU-Q47) designed to measure health literacy in adults. The HLS-EU-Q47 has been widely validated internationally [29]. The HLS_19_-Q12 was developed as a shorter measure and reflects recent definitions of health literacy. HLS_19_-Q12 response options include 1 = very difficult, 2 = difficult, 3 = easy, 4 = very easy. The total score is calculated as the mean of the numeric values of item responses, scaled from 0 to 100 [29]. Higher scores reflect better health literacy. Levels of health literacy have been categorized as excellent (>83.33), sufficient (>66.67 and ≤83.33), problematic (>50 and ≤66.67) and inadequate (≤50), based on normative cut-offs from the HLS-EU-Q47 [29]. These cut-off values were determined based on expert assessments and statistical testing to maximize the correlation between categorical levels of health literacy and corresponding continuous scores as well as minimizing the influence of covariates on these scores [30]. The HLS_19_-Q12 has been shown to correlate well with corresponding scores on the HLS-EU-Q47 [29] and demonstrate adequate internal consistency and validity across countries and languages in adult populations (mean α = 0.78) [31]. Results from confirmatory factor analysis demonstrated good model fit across 17 countries, suggesting the HLS_19_-Q12 is valid for measuring health literacy across countries. Evidence was found for differential item functioning for some items for some countries, although their influence on total scores was not evaluated [29].

### Statistical analysis

We computed descriptive statistics for all variables. For health literacy, we reported the mean and standard deviation of the percentage of items with valid responses that were answered with ‘very easy’ or ‘easy’. We also reported frequencies with percentages of participants in the excellent, sufficient, problematic and inadequate health literacy categories [29]. Simple linear regressions were used to assess bivariate associations between sociodemographic and medical variables with health literacy. Multivariable linear regression was used to assess the independent association of each variable with health literacy. We identified items to be included in the model *a priori* based on previous studies of factors associated with health literacy in general and major disease populations [10–16]. Variables included in the main analysis were age, gender, race or ethnicity, education, marital status, country, location of residence, time since first non-Raynaud’s symptom and disease subtype. Australia, Mexico and Spain were grouped into a single ‘other countries’ variable because there were insufficient participants to analyse separately (Australia *N* = 18, Mexico *N* = 5, Spain *N* = 21).

We accounted for missing data by using multiple imputation via chained equations, using the mice package in R [32]. We generated 20 imputed datasets, using 15 cycles per dataset. Variables included in the mice procedure were all variables in the main regression model and additional disease and sociodemographic variables measured in the SPIN Cohort: smoking status, alcohol consumption, body mass index and the physical function, anxiety, depression, pain intensity, pain interference, fatigue, sleep and satisfaction with social roles and activities domain scores from the PROMIS-29v2.0 [33].

We conducted three sensitivity analyses. First, we analysed only participants with complete data. Second, we used sex rather than gender in the main analysis. Third, we analysed data from participants who took part in both the present study and our earlier study on factors associated with eHealth literacy in SSc [21] (*N* = 283 participants completed both) and compared eHealth literacy and health literacy results.

We reported unstandardized regression coefficients with 95% confidence intervals (CIs) and total explained variance for each model (adjusted *R^2^*). All regression analyses were conducted in R (R version 3.6.3, RStudio Version 1.2.5042). Because we observed a strong association between health literacy and country and a small association between health literacy and education, *post hoc* we calculated Pearson correlations between health literacy and education stratified by country.

## Results

There were 1019 participants from 46 sites who completed the HLS_19_-Q12 and were included. As shown in Table 1, participants were predominantly female (*N* = 898, 88%) and White (*N* = 850, 84%). The mean (standard deviation [SD]) age was 62.4 years (SD = 11.7), and mean education was 15.5 years (SD = 3.9). Most participants were married or living as married (*N* = 701, 69%); from France (*N* = 391, 38%), the USA (*N* = 275, 27%) or Canada (*N* = 228, 22%); and living in cities (*N* = 355, 35%), suburban areas (*N* = 271, 27%), villages or towns (*N* = 261, 26%) or rural areas (*N* = 108, 11%). Mean years since first non-Raynaud phenomenon symptom was 17.2 (SD = 9.8) and most people had limited cutaneous SSc or sine scleroderma (*N* = 669, 66%).

**Table 1:** Sample sociodemographic and disease characteristics in 1019 people with systemic sclerosis.

	N with data	Mean (SD) or N (%)
Age (years)	1019	62.4 (11.7)
Female sex	1019	898 (88.1%)
Gender	1019	
Cisgender woman		864 (84.8%)
Cisgender man		117 (11.5%)
Other		38 (3.7%)
White race or ethnicity^a^	1017	850 (83.6%)
Education (years)	1006	15.5 (3.9)
Education >12 years	1006	765 (76.0%)
Marital status	1011	
Married or living as married		701 (69.3%)
Divorced or separated		132 (13.1%)
Single		111 (11.0%)
Widowed		67 (6.6%)
Country	1019	
France		391 (38.4%)
USA		275 (27.0%)
Canada		228 (22.4%)
UK		81 (7.9%)
Australia, Mexico or Spain^b^		44 (4.3%)
Location	1002	
City		355 (35.4%)
Suburb		271 (27.0%)
Village or town		261 (26.0%)
Rural		108 (10.8%)
Other^c^		7 (0.7%)
Years since first non-Raynaud phenomenon symptom	943	17.2 (9.8)
Diffuse cutaneous disease subtype^d^	1013	344 (34.0%)
Health Literacy Scale_19_ − 12 Item Questionnaire score	1019	69.7 (16.8)
Excellent (>83.33)		247 (24.2%)
Sufficient (>66.67 and ≤83.33)		191 (18.7%)
Problematic (>50 and ≤66.67)		465 (45.6%)
Inadequate (≤50)		116 (11.4%)

a Race or ethnicity data are self-reported in each country using standard categories used in each country. Therefore, categories differed between countries.

b *N* = 18 from Australia, *N* = 5 from Mexico, *N* = 21 from Spain.

c Other response included responses not able to be classified in existing categories.

d Versus limited SSc (*N* = 631) or sine SSc (*N* = 38).

N: number; SD: standard deviation; SSc: systemic sclerosis.

The mean HLS_19_-Q12 total score was 69.7 (SD = 16.8). There were 247 (24%) participants classified as having excellent health literacy, 191 (19%) with sufficient health literacy, 465 (46%) with problematic health literacy and 116 (11%) with inadequate health literacy.

In the main multivariable analyses (Table 2), higher education (1.63 points per 5 years, 95% CI 0.25–3.01) was significantly associated with higher health literacy scores. Among countries, participants living in France (−6.05 points, 95% CI −8.84 to −3.27), Canada (−4.98 points, 95% CI −7.99 to −1.97), the UK (−7.79 points, 95% CI −12.03 to −3.55) and Australia, Mexico or Spain (−7.02 points, 95% CI −12.39 to −1.66) had statistically significantly lower health literacy scores compared with participants living in the USA. Age, gender, race or ethnicity, marital status, location, years since first non-Raynaud phenomenon symptom and diffuse cutaneous subtype were not associated with health literacy.

**Table 2:** Linear regression analysis of sociodemographic and disease characteristic associations with health literacy (*N* = 1019).

	Bivariate (unadjusted) regression coefficient (95% CI)	Multivariable (adjusted) regression coefficient (95% CI)
Age (years)	0.07 (−0.02, 0.15)	0.06 (−0.03, 0.15)
Gender (reference = woman)		
Man	0.42 (−2.83, 3.67)	0.30 (−2.95, 3.56)
Other	−0.45 (−5.92, 5.02)	0.39 (−5.04, 5.81)
Race or ethnicity other than White (reference = White)	−2.42 (−5.20, 0.37)	−2.38 (−5.19, 0.44)
Education (per 5 years)	**2.25 (0.91, 3.58)**	**1.63 (0.25, 3.01)**
Single, divorced or separated, widowed (reference = married or living as married)	−0.04 (−2.29, 2.21)	−0.01 (−2.24, 2.23)
Country (reference = USA)		
France	−**7.32 (**−**9.87,** −**4.77)**	−**6.05 (**−**8.84,** −**3.27)**
Canada	−**5.39 (**−**8.29,** −**2.49)**	−**4.98 (**−**7.99,** −**1.97)**
UK	−**9.20 (**−**13.30,** −**5.11)**	−**7.79 (**−**12.03,** −**3.55)**
Australia, Mexico or Spain	−**7.62 (**−**12.88,** −**2.36)**	−**7.02 (**−**12.39,** −**1.66)**
Location (reference = city)		
Village or town	−**2.68 (**−**5.35,** −**0.01)**	−2.20 (−4.99, 0.60)
Suburb	2.46 (−0.19, 5.10)	0.90 (−1.81, 3.61)
Rural	1.79 (−1.82, 5.40)	0.57 (−3.03, 4.18)
Other	−0.04 (−12.56, 12.48)	1.82 (−10.62, 14.27)
Years since first non-Raynaud phenomenon symptom	0.01 (−0.10, 0.12)	−0.03 (−0.14, 0.09)
Diffuse cutaneous subtype (reference = limited or sine)	0.41 (−1.77, 2.59)	−0.60 (−2.84, 1.64)

Adjusted R^2^ = 0.05. Statistically significant coefficients are bolded.

CI: confidence interval.

Complete case analysis results, which included 917 participants, were similar to results from the main analysis (see Supplementary Table S1). Substituting sex for gender in the main analysis did not affect results (see Supplementary Table S2). When we included only the 283 participants who also participated in our study on factors associated with eHealth literacy in SSc, results were similar to the main analysis (see Supplementary Table S3).

The Pearson correlation between education in years and health literacy score for the total sample was 0.11. Pearson correlations between education in years and health literacy score were between 0.01 and 0.05 for Canada, the USA and the UK; 0.15 for France; and −0.15 for other countries (see Supplementary Table S4).

## Discussion

We found that 43% of participants had excellent or sufficient health literacy scores and 57% had problematic or inadequate scores. Higher education was statistically significantly associated with higher health literacy scores, but the magnitude was minimal to small [34]. Health literacy scores were about one-tenth of a standard deviation greater per 5 years of additional education. There is no established minimally important difference for HLS_19_-Q12 scores. However, given health literacy category ranges of excellent (>83.33), sufficient (>66.67 and ≤83.33), problematic (>50 and ≤66.67), or inadequate (≤50), the difference we observed should be considered minimal and not likely clinically meaningful. Participants from France, Canada, the UK and other countries (Australia, Mexico or Spain) had statistically significantly lower reported health literacy than participants from the USA, with magnitudes typically considered small to medium effects (about one-third to one-half of a standard deviation) [34]. No other sociodemographic or disease factors were statistically significantly associated with health literacy.

The small positive association of education with health literacy is surprising and appeared to be almost entirely due to France (Pearson correlation = 0.15) and not present in other countries (Pearson correlations = 0.01–0.05). It is often reported or assumed that health literacy and education are highly related, but there are other examples where correlations between self-reported health literacy and education are small and some evidence that the relationship between self-reported health literacy and education may be tenuous [11, 35–37]. For example, a comparative survey of health literacy across eight European countries using the HLS-EU-Q47 indicated education had only a small significant correlation with health literacy (Pearson correlation = 0.25), whereas financial deprivation had the strongest association (Pearson correlation = −0.34) followed by perceived social status (Pearson correlation = 0.31) [11]. These findings support the presence of a social gradient in health literacy, accounted for only weakly by education. Further, a study on health literacy among adults in the Netherlands (*N* = 925) found education was not associated with all health literacy domains as measured by the HLS-EU-Q47 [37]; education was significantly associated with health literacy for accessing and understanding information, but not for appraising and applying information [37].

The mean HLS_19_-Q12 score in our sample was 69.7 (SD = 16.8), which is slightly higher than HLS_19_-Q12 scores reported in other samples from SPIN Cohort countries, including a general population sample from France (*N* = 2003, mean = 65.9, SD = 15.5) [29, 38] and health service users in Spain (*N* = 150, mean = 66.0, SD = 13.6) [39]. By country, participants from the USA rated themselves higher on health literacy than people from France, Canada and the UK. One interpretation might be related to differences in health systems. People in the USA are required to navigate complex health care systems, insurance plans, coverage and costs [40], whereas France, Canada, the UK and Australia, Mexico and Spain have universal healthcare systems that endeavour to provide more accessible medical care [41]. Nevertheless, results across other studies suggest inconsistencies between countries that may preclude drawing easy conclusions. For instance, in the main development and validation paper of the HLS_19_-Q12 [29], which reports results from 17 European countries, people from Germany had the lowest mean health literacy score (mean = 58.9, SD = 13.6; total mean across countries = 64.7, range = 58.9–72.3), despite having one of the most highly educated populations in the world [42]. Among people from Portugal, the mean health literacy score was close to the total European average (mean = 63.8, SD = 11.5) [29], even though Portugal has one of the lowest levels of formal educational attainment in Europe (45.5% people with less than secondary education compared with a 22.2% European country mean) [42].

It is also possible that how people perceive and report their own health literacy is influenced by cultural and social factors not directly related to the construct of health literacy. For instance, numerous studies have found that people from the USA score higher on patient-reported outcome measures than other countries, potentially due to stronger cultural tendencies to rate themselves positively on self-report scales irrespective of objective health status [43]. Further, a report on health literacy and self-reported health across 23 countries found cross-country variations in the correlation between years of schooling, health literacy and self-reported health, indicating associations are context dependent; other factors such as health equity, social cohesion and social norms may be more central [44]. Measures of objective health literacy (e.g. numeracy, reading comprehension skills) and subjective health literacy (e.g. perceived ability to appraise and apply information) are based on distinct theoretical constructs [45]. More attention to how we study health literacy and define measurements is needed to be able to confidently interpret and apply results. Whether the HLS_19_-Q12 and its longer forms are measuring an ability to access, understand, appraise and apply health information, as theoretically assumed, or other influences such as cultural norms, is not well understood.

Our finding that health literacy had a minimal to small association with education differs somewhat from a previous study in the SPIN Cohort (*N* = 333) on eHealth literacy [21], which found that education was not associated with eHealth literacy (−0.05 points per 5 years, 95% CI −0.80–0.70). In a sensitivity analysis of the present study, we evaluated 283 people who participated in both studies and found the association between education and health literacy was similar to our main analysis (*N* = 1019). The somewhat different associations of education with health literacy and eHealth literacy may be due to how these concepts are defined and measured. Unlike health literacy, eHealth literacy measures are designed to assess constructs related to health information found on the internet, which requires an ability to navigate the internet, use new technologies and effectively search for information, rather than obtaining, understanding and making decisions based on health information more generally [22]. More work is needed to clarify these relationships.

The adjusted R^2^ for our main multivariable regression model was 0.05. This may appear low, but it is expected in samples comprised entirely of people with a chronic condition as all have the similar experience of living with the condition. High R^2^ values are important in predictive modelling, but much less so when models are used for testing hypotheses about possible associations of variables of interest with critical patient-important outcomes. In this case, including in the present study, having a sufficiently large sample size to generate reasonably precise parameter estimates is a more important consideration [46].

### Study strengths and limitations

Our study had important strengths. First, 10 people with lived experience of SSc were engaged as research partners through membership on the SPIN Steering Committee, which prioritised this work. They are also co-authors of this manuscript and will be involved in dissemination of the results to the SSc community through co-development of research dissemination tools. Second, participants were recruited from a large, multinational cohort of people with SSc from seven countries, which provided a large sample size and allowed us to explore differences in health literacy by country.

There are also limitations to consider. First, the cross-sectional study design does not allow us to infer causal relationships based on our results. Second, whilst the HLS_19_-Q12 has been validated internationally, full measurement invariance across countries has not been established [29]. Therefore, differences in health literacy between countries should be interpreted with caution. Third, SPIN Cohort participants are recruited from specialty SSc centres in highly developed countries and have a high mean level of formal education, both of which might reduce generalizability of results. Fourth, SPIN Cohort participants complete outcome measures every three months via the internet and may participate in other SPIN programs to test patient-oriented interventions or measures. This could possibly improve health literacy through increased exposure to health information and improved knowledge or understanding of health information. Thus, self-reported levels of health literacy among participants in this study may be higher than the broader population of people with SSc. Finally, we carefully selected sociodemographic variables measured in our cohort that were suggested as potentially important in the literature. It is possible other important variables were excluded, which may have resulted in overestimation of observed relationships.

## Conclusion

We found that education had a minimal to small association with health literacy among people with SSc. People with SSc from France, Canada, the UK and Australia, Mexico or Spain had significantly lower self-reported health literacy than people from the USA. The minimal association between education and health literacy differs from theoretical expectations but is consistent with other studies that have reported similar results. It is possible that the country differences we identified could reflect reporting tendencies related to self-evaluation. More research on how we define and measure health literacy is needed to confidently interpret and apply findings.

### Supplementary material

Supplementary material is available at *Rheumatology* online.

## Supplementary Material

keag558_Supplementary_Data

## Data Availability

The data underlying this article will be shared on reasonable request to the corresponding author.
