# Supplementary material for: Health literacy among people with systemic sclerosis: a Scleroderma Patient-Centred Intervention Network (SPIN) Cohort cross-sectional study
Source: Rheumatology (Oxford). 2026 Oct 19;65(10):keag558. doi: 10.1093/rheumatology/keag558 (PMC13711234; doi:10.1093/rheumatology/keag558)
Supplement: Supplementary file 1 [file keag558_supplementary_data.docx]

**SUPPLEMENTARY MATERIAL**

**Table of Contents**

**Supplementary Table S1.** Complete Case Linear Regression Analysis of Sociodemographic and Disease Characteristic Associations with Health Literacy (N = 917) (p 2)

**Supplementary Table S2.** Linear Regression Analysis of Sociodemographic and Disease Characteristic Associations with Health Literacy, substituting Sex for Gender (N = 1,019) (p 3)

**Supplementary Table S3.** Comparison of Multivariable Analyses of Sociodemographic and Disease Characteristic Associations with Health Literacy in Full Sample and Among People who Participated in Both the Present Study and Previous Study on eHealth Literacy (p 4)

**Supplementary Table S4.** Pearson Correlations between Education Years and Health Literacy, in Total Sample and by Country (N = 1,006) (p 5)

**Supplementary Table S1.** Complete Case Linear Regression Analysis of Sociodemographic and Disease Characteristic Associations with Health Literacy (N = 917)

|  | **Bivariate (Unadjusted) Regression Coefficient** **(95% CI)** | **Multivariable (Adjusted) Regression Coefficient** **(95% CI)** |
| --- | --- | --- |
| Age (years) | 0.07 (-0.02, 0.15) | 0.04 (-0.06, 0.14) |
| Gender (reference = woman) |  |  |
| Man | 0.42 (-2.83, 3.67) | 0.02 (-3.38, 3.42) |
| Other | -0.45 (-5.92, 5.02) | -1.02 (-6.97, 4.93) |
| Non-White (reference = White) | -2.42 (-5.21, 0.37) | -2.49 (-5.51, 0.52) |
| Education (per 5 years) | **2.28 (0.96, 3.60)** | **1.56 (0.12, 2.99)** |
| Single, divorced or separated, widowed (reference = married or living as married) | 0.02 (-2.23, 2.26) | 0.25 (-2.12, 2.62) |
| Country (reference = United States) |  |  |
| Canada | **-5.39 (-8.29, -2.49)** | **-4.82 (-7.99, -1.66)** |
| United Kingdom | **-9.20 (-13.30, -5.11)** | **-7.63 (-12.40, -2.91)** |
| France | **-7.32 (-9.87, -4.77)** | **-5.82 (-8.79, -2.85)** |
| Australia, Mexico, or Spain | **-7.62 (-12.90, -2.36)** | **-6.51 (-12.10, -0.96)** |
| Location (reference = city) |  |  |
| Village or town | **-2.67 (-5.35, 0.01)** | -2.40 (-5.38, 0.58) |
| Suburb | 2.53 (-0.12, 5.18) | 1.06 (-1.77, 3.89) |
| Rural | 1.88 (-1.72, 5.49) | 0.28 (-3.54, 4.09) |
| Other | 0.00 (-12.50, 12.50) | 1.86 (-11.60, 15.40) |
| Years since first non-Raynaud phenomenon symptom | 0.01 (-0.10, 0.12) | -0.02 (-0.13, 0.10) |
| Diffuse cutaneous subtype (reference = limited or sine) | 0.41 (-1.78, 2.60) | -0.15 (-2.52, 2.21) |

CI = confidence interval

Adjusted R^2^ = 0.03. Statistically significant coefficients are bolded.

**Supplementary Table S2.** Linear Regression Analysis of Sociodemographic and Disease Characteristic Associations with Health Literacy, substituting Sex for Gender (N = 1,019)

|  | **Bivariate (Unadjusted) Regression Coefficient** **(95% CI)** | **Multivariable (Adjusted) Regression Coefficient** **(95% CI)** |
| --- | --- | --- |
| Age (years) | 0.07 (-0.02, 0.15) | 0.06 (-0.04, 0.15) |
| Sex (reference = female) | 0.67 (-2.52, 3.87) | 0.68 (-2.54, 3.89) |
| Non-White (reference = White) | -2.42 (-5.20, 0.37) | -2.38 (-5.19, 0.44) |
| Education (per 5 years) | **2.25 (0.91, 3.58)** | **1.63 (0.26, 3.01)** |
| Single, divorced or separated, widowed (reference = married or living as married) | -0.04 (-2.29, 2.21) | 0.01 (-2.22, 2.25) |
| Country (reference = United States) |  |  |
| Canada | **-5.39 (-8.29, -2.49)** | **-4.97 (-7.97, -1.96)** |
| United Kingdom | **-9.20 (-13.30, -5.11)** | **-7.76 (-12.00, -3.53)** |
| France | **-7.32 (-9.87, -4.77)** | **-6.05 (-8.83, -3.27)** |
| Australia, Mexico, or Spain | **-7.62 (-12.88, -2.36)** | **-7.00 (-12.36, -1.63)** |
| Location (reference = city) |  |  |
| Village or town | **-2.68 (-5.35, -0.01)** | -2.22 (-5.01, 0.56) |
| Suburb | 2.46 (-0.19, 5.10) | 0.88 (-1.82, 3.58) |
| Rural | 1.79 (-1.82, 5.40) | 0.55 (-3.05, 4.16) |
| Other | -0.04 (-12.56, 12.48) | 1.88 (-10.55, 14.31) |
| Years since first non-Raynaud phenomenon symptom | 0.01 (-0.10, 0.12) | -0.02 (-0.14, 0.09) |
| Diffuse cutaneous subtype (reference = limited or sine) | 0.41 (-1.77, 2.59) | -0.64 (-2.88, 1.60) |

CI = confidence interval

Adjusted R^2^ = 0.05. Statistically significant coefficients are bolded.

**Supplementary Table S3.** Comparison of Multivariable Analyses of Sociodemographic and Disease Characteristic Associations with Health Literacy in Full Sample and Among People who Participated in Both the Present Study and Previous Study on eHealth Literacy

|  | **Main**  **Multivariable (Adjusted) Regression Coefficient** **(95% CI)**  **(N = 1,019)** | **Sub-group**^a^ **Multivariable (Adjusted) Regression Coefficient** **(95% CI)**  **(N = 283)** |
| --- | --- | --- |
| Age (years) | 0.06 (-0.03, 0.15) | -0.02 (-0.20, 0.16) |
| Gender (reference = woman) |  |  |
| Man | 0.30 (-2.95, 3.56) | -0.64 (-6.98, 5.70) |
| Other | 0.39 (-5.04, 5.81) | **16.01 (2.09, 29.94)** |
| Non-White (reference = White) | -2.38 (-5.19, 0.44) | -0.95 (-6.48, 4.57) |
| Education (per 5 years) | **1.63 (0.25, 3.01)** | 1.43 (-1.23, 4.09) |
| Single, divorced or separated, or widowed (reference = married or living as married) | -0.01 (-2.24, 2.23) | 1.35 (-2.98, 5.69) |
| Country (reference = United States) |  |  |
| Canada | **-4.98 (-7.99, -1.97)** | **-5.64 (-11.13, -0.15)** |
| United Kingdom | **-7.79 (-12.03, -3.55)** | **-9.71 (-17.73, -1.68)** |
| France | **-6.05 (-8.84, -3.27)** | -3.22 (-8.69, 2.26) |
| Australia, Mexico, or Spain | **-7.02 (-12.39, -1.66)** | -1.65 (-13.96, 10.67) |
| Location (reference = city) |  |  |
| Village or town | -2.20 (-4.99, 0.60) | 1.13 (-4.41, 6.67) |
| Suburb | 0.90 (-1.81, 3.61) | -0.47 (-5.55, 4.62) |
| Rural | 0.57 (-3.03, 4.18) | 5.07 (-2.03, 12.17) |
| Other | 1.82 (-10.62, 14.27) | 7.66 (-15.76, 31.07) |
| Years since first non-Raynaud phenomenon symptom | -0.03 (-0.14, 0.09) | 0.01 (-0.20, 0.22) |
| Diffuse cutaneous subtype (reference = limited or sine) | -0.60 (-2.84, 1.64) | 0.14 (-4.00, 4.28) |

CI = confidence interval

Adjusted R^2^ for sub-group analysis = 0.06. Statistically significant coefficients are bolded.

**Supplementary Table S4.** Pearson Correlations between Education Years and Health Literacy, in Total Sample and by Country (N = 1,006)

| **Country** | **N** | **Pearson correlation coefficient** |
| --- | --- | --- |
| Canada | 227 | 0.01 |
| United States | 272 | 0.04 |
| United Kingdom | 81 | 0.05 |
| France | 382 | 0.15 |
| Australia, Mexico, or Spain | 44 | -0.15 |
| **Total** | **1006** | **0.11** |
